# Beta-hydroxybutyrate (BHB) elicits concentration-dependent anti-inflammatory effects on microglial cells which are reversible by blocking its monocarboxylate (MCT) importer

**DOI:** 10.3389/fragi.2025.1628835

**Published:** 2025-07-29

**Authors:** Chase Garcia, Ariana Banerjee, Claire Montgomery, Lauren Adcock, Izumi Maezawa, Jon Ramsey, Ana Cristina G. Grodzki, Kyoungmi Kim, Gino Cortopassi

**Affiliations:** ^1^ Department of Molecular Biosciences, School of Veterinary Medicine, University of California at Davis, Davis, CA, United States; ^2^ Department of Pathology and Laboratory Medicine, UC Davis Medical Center, University of California at Davis, Sacramento, CA, United States; ^3^ Department of Public Health Sciences, School of Medicine, University of California at Davis, Davis, CA, United States

**Keywords:** Alzheimer’s, microglia, ketone, beta-hydroxybutyrate, TREM2, BHB, Alzheimer’s disease, ketogenic diet

## Abstract

**Introduction:**

The ketogenic diet (KD) increases mouse lifespan and health span, and improves late-life memory. This raises the question regarding the mechanism behind this effect. In mice on a KD, blood beta-hydroxybutyrate (BHB) levels uniquely rise higher than those of mice on a control diet (CD). BHB is therefore considered a key signaling and metabolic mediator of KD’s effects and benefits. BHB crossed the blood–brain barrier and rescued memory, improved cognitive function, and increased neuronal plasticity in two different mouse models of Alzheimer’s disease (PS1/APP and 5XFAD). At the cellular level, microglia are thought to play a critical role in the physiologic basis of memory due to their important role in synaptic development, plasticity, and connectivity. Conversely, microglial dysfunction and inflammation are connected to cognitive decline and neurodegenerative diseases. Because of this, one explanatory hypothesis for these positive therapeutic observations in mice is that the KD and BHB drive memory and longevity benefits through their anti-inflammatory actions on microglia.

**Method:**

We investigated the concentration dependence of BHB’s antiinflammatory effects in BV2 microglial cells. We focused on 1.5 mM BHB, which reflects blood levels in mice and humans on a KD.

**Results:**

At this concentration, BHB significantly and concentration-dependently decreased the following: 1) inflammatory cytokine expression (IL-6, TNF-α, and IL-1β), 2) inflammatory morphological changes, and 3) activation of p-ERK and p-p38MAPK, which are key pathways involved in microglial inflammation. We show, for the first time, that the expression of Alzheimer’s risk gene TREM2 is modified by dietarily-achievable 1.5 mM BHB. BHB’s anti-inflammatory, morphological, biochemical, and TREM2 effects were blocked by a monocarboxylate transporter (MCT) inhibitor, supporting the idea that BHB must enter microglia to elicit some of its anti-inflammatory effects.

**Discussion:**

These results support the hypothesis that blood BHB levels achievable on a KD elicit significant concentration-dependent anti-inflammatory effects in microglia. Increasing BHB concentration through sustained KD, or BHB supplements, may lower microglial inflammatory tone and provide benefits in age-related memory loss.

## Graphical Abstract

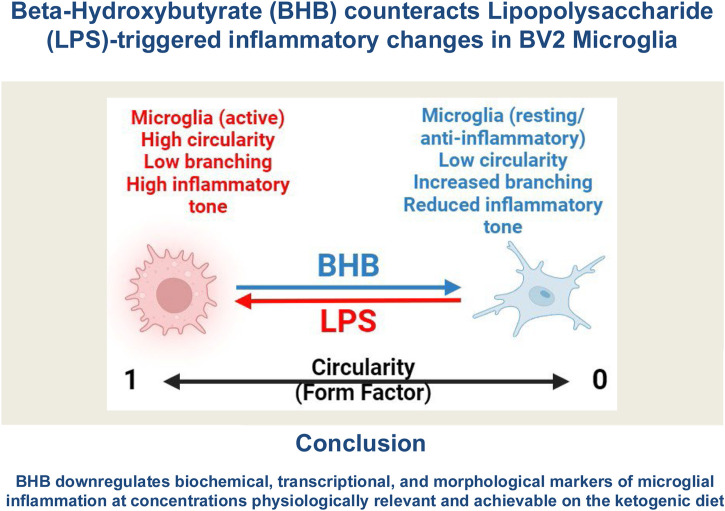

## Introduction

Alzheimer’s disease (AD) is an age-related irreversible neurodegenerative disease hallmarked by amyloid-beta (Aβ) plaque pathologies and typified by clinical signs of memory loss, confusion, and cognitive decline (Knopman et al., 2021). AD affected over 7 million Americans in 2024, accounting for 70% of all dementia cases, and is the fifth leading cause of death in the United States (Alzheimer’s Association, 2024). Despite >30 years of intense research and therapeutic development, effective therapies are limited.

Microglial dysfunction is related to cognitive decline in aging and AD (Gao et al., 2023). Microglia are the resident innate immune cells of the brain and respond to local injury and infection (Paolicelli et al., 2011). Importantly to AD, microglia also regulate synaptic development and long-term potentiation (LTP), which is a cellular marker of memory, connectivity, and plasticity (Paolicelli et al., 2011; Andoh and Koyama, 2021; Nguyen et al., 2020). Several recent papers support the concept that microglial function has a significant impact on AD risk and progression. Genetic data support that many AD risk factors are highly and selectively expressed in microglia (Hansen et al., 2018; Sala Frigerio et al., 2019). It has been suggested that microglial hyper-inflammation and overactive synaptic pruning may decrease synaptic plasticity in AD, suppress new memory formation, and contribute to AD symptoms (Hong et al., 2016; Vanderheyden et al., 2018; Brucato and Benjamin, 2020). Additionally, cytokines secreted from inflamed microglia may contribute to AD pathophysiology too (Wang WY. et al., 2015; Tanaka et al., 2014; Lyra et al., 2021).

Dysregulation of the microglial inflammatory tone may contribute to neurodegenerative diseases (Guo et al., 2022). Aging and other AD risk factors may converge on microglia to drive an aberrant, chronic, inflammatory state (Guo et al., 2022). This inflamed state may impair normal microglial and brain function, reduce clearance of amyloid plaques, and drive AD (Hansen et al., 2018; Wang WY. et al., 2015; Guo et al., 2022; Bernier et al., 2020; Ghosh et al., 2018; Dutta et al., 2023). Identifying nutritional or supplement strategies to reduce age- or AD-dependent microglial inflammation could have therapeutic benefits (Paidi et al., 2023).

A ketogenic diet (KD) is a diet that is very low in carbohydrates. In humans, a KD often contains less than 35 g of carbohydrates per day, whereas for mice, a KD contains ∼1% of the total kcal as carbohydrates (Zhou et al., 2023; Di Lucente et al., 2024a; Roberts et al., 2017). Consuming a KD causes a physiological increase in blood BHB. In mice on a KD, BHB is sustainably maintained at ∼1.5 mM. BHB levels of 1.5 mM are never sustainably achieved on an *ad libitum* control diet (CD) (Zhou et al., 2023; Di Lucente et al., 2024a; Roberts et al., 2017; Pathak et al., 2022; Newman et al., 2017). With good compliance, a KD can increase blood [BHB] in humans up to ∼2 mM (Newman and Verdin, 2017). On a KD, BHB functions as both a metabolic alternative to glucose and a potent signaling molecule that crosses the blood–brain barrier and can modulate microglial function (Newman and Verdin, 2017; Sun et al., 2023; González Ibáñez et al., 2023).

We and others previously showed that the KD, a diet extremely low in carbohydrates, extended longevity and improved late-life memory and cognition in aged C57Bl6 mice (Roberts et al., 2017; Newman et al., 2017). We previously analyzed blood BHB levels in aging C57Bl6 mice that experience memory loss and found that BHB levels were twice as high in mice on a KD compared to mice on a CD (Roberts et al., 2017; Newman et al., 2017), and only KD mice with 2X higher BHB levels experienced a significant memory benefit compared to CD^24^ mice. Similar results were seen when we and others tested BHB directly in the 5XFAD AD mouse model (Wu et al., 2020; Di Lucente et al., 2024b). These data suggest that increasing BHB levels may support increased memory, which leads to the question of how this might occur.

Recent *in vitro* and *in vivo* evidence suggests that BHB reduces microglial inflammation, and this is what leads to memory benefit in the AD context. BHB changed microglial morphology toward a nonreactive phenotype and increased transcription of anti-inflammatory cytokines (Polito et al., 2023; Zhang et al., 2022; Huang et al., 2018). We recently observed that BHB concentration-dependently suppressed human microglial inflammation and phagocytic dysfunction provoked by Aβ oligomer, and this suppression was more potent at 1.5 mM than at 0.1 mM^33^. Furthermore, we showed that 3 mM BHB applied directly to the hippocampi from PS1/APP mice, which are a mouse model of AD, rescued LTP (Di Lucente et al., 2024a). Thus, as microglia control synaptic plasticity and memory formation (Hong et al., 2016; Vanderheyden et al., 2018; Brucato and Benjamin, 2020), and respond to lipids and their derivatives, which include ketones (Wang Y. et al., 2015), it is our hypothesis that BHB, through its *concentration-dependent* anti-inflammatory effect in microglia, preserves memory at BHB concentrations that are *only* sustainably achieved on the KD (BHB = 1.5 mM) but not on a control diet in which the BHB levels are much lower.

In the current study, we focus on whether BHB’s positive therapeutic effects are *concentration dependent* and aim to identify the therapeutic range of this action. We chose to study the anti-inflammatory potency of BHB in the well-characterized BV2 microglial cell line. For the reasons stated above, in this study, we focused on the concentration-dependent effects of BHB from the low physiological blood BHB concentration range observed in *ad libitum*-fed mice and people on a CD, that is, ∼0.5 mM BHB to the high physiological BHB concentration range achieved sustainably on a KD, that is, ∼1.5 mM.

The results of our study support the view that BHB is concentration-dependently anti-inflammatory for mouse microglia and provide a potential clue as to why the KD increases synaptic plasticity and memory in the mouse models of AD PS1/APP (Di Lucente et al., 2024a) and 5XFAD (Wu et al., 2020; Di Lucente et al., 2024b). These results support the view that the KD, through its production of BHB, acts as an anti-inflammatory drug, and that the concentration of BHB controls its anti-inflammatory potency.

## Results

### Lipopolysaccharide (LPS) and BHB significantly altered microglial BV2 cell circularity

**FIGURE 1:**
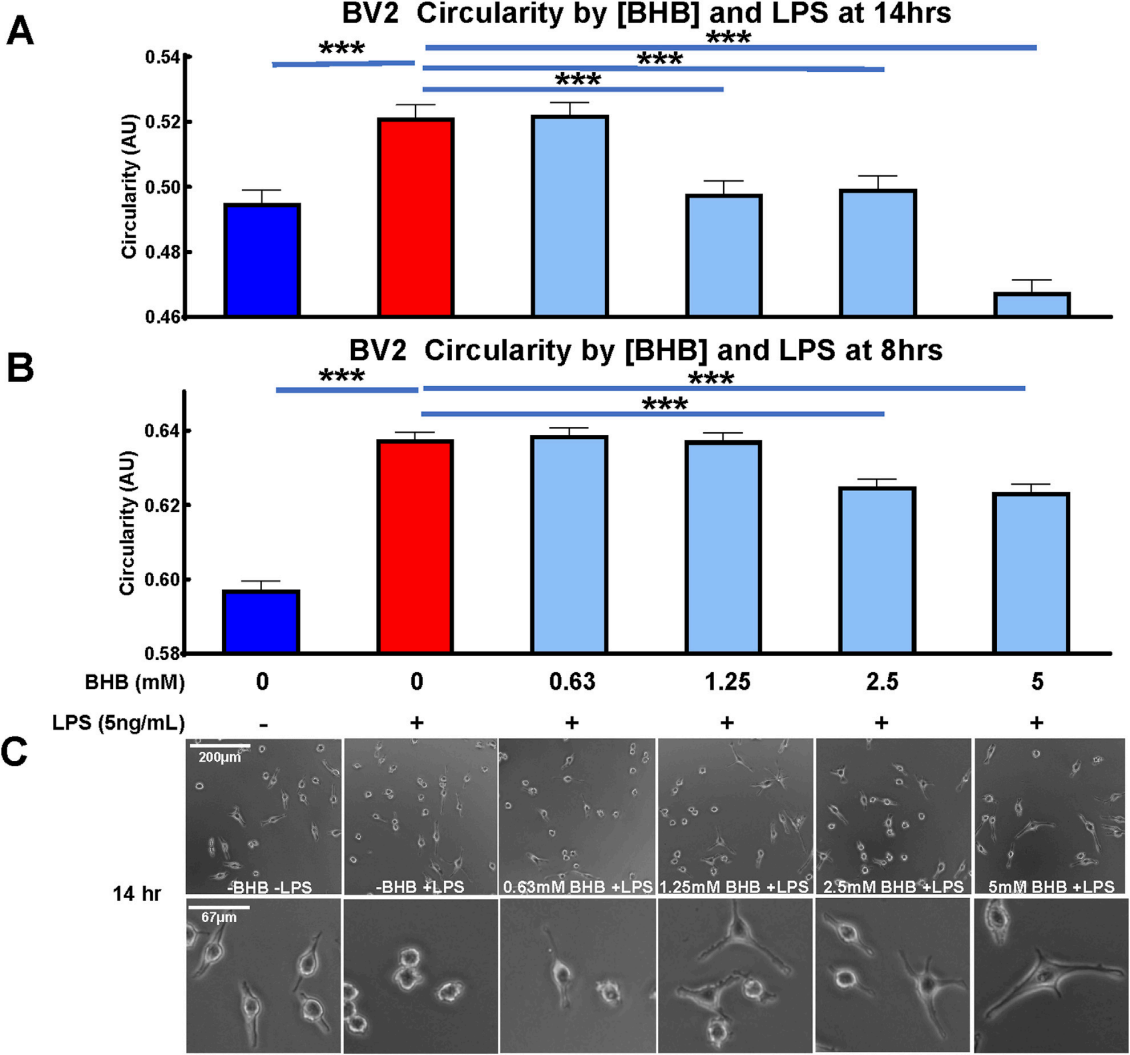
Beta-hydroxybutyrate (BHB) significantly decreased circularity in BV2 microglia in a concentration- and time-dependent manner. Circularity compares cell area to the perimeter on a scale of 0–1. Homeostatic/anti-inflammatory microglia are associated with low-circularity morphology, i.e., closer to 0. Cells were plated, incubated overnight, then supplemented with lipopolysaccharide (LPS) and BHB, and incubated for a specified time before imaging. **(A, B)** These data show that BHB concentration- and time-dependently decreased circularity at 8 **(B)** and 14 **(A)** hours after supplementing with increasing titration of BHB and 5 ng/mL LPS, suggesting that BHB promoted an anti-inflammatory phenotype in these microglia. These metrics were quantified from Calcein AM-derived fluorescent images captured by BioTek Cytation5 imager. Calcein AM only stains live cells, and thus, the changes presented here do not reflect changes in cell viability. An automated and unbiased thresholding process in Gen5 software application was used to outline cells and quantify the perimeter and area from these images. Bars are the mean +SEM. Each bar represents an average of ∼7,000 individual cell measurements. For statistical analysis, one-way ANOVA was used for a linear trend test on groups treated with LPS (p < 0.0001 for 8 h and 14 h time-points), followed by *post hoc* Dunnett’s tests for multiple comparisons, which compared baseline control and BHB exposure groups to the [LPS] control. Asterisks represent significance values from the *post hoc* Dunnett’s test. * = p < 0.05, ** = p < 0.01, and *** = p < 0.001. **(C)** Representative images of each group from **(A)** data, i.e. 14 h time-point, and they include two levels of magnification for images.

The potent inflammatory immunogen LPS, also known as endotoxin, induced BV2 microglia to assume a circular morphology (Figures 1A, B) that had minimal branching (Figure 1C), and these observations are consistent with inflamed microglial morphology (Vidal-Itriago et al., 2022). At two time-points after BHB and LPS administration, at 8 h and 14 h, BHB concentration-dependently decreased microglial circularity. At 8 h, 2.5–5 mM BHB significantly reduced circularity. As the BHB concentration increased, the LPS-induced circular phenotype became more branched; at the highest BHB concentrations, microglia adopted a more branched and elongated phenotype, like the untreated control [-BHB -LPS]. The effect of LPS is completely reversed at the 14 h time-point, at which 1.25 mM and 2.5 mM BHB completely and significantly rescued LPS-induced circular microglial morphology. BHB at the concentration of 5 mM reduced circularity past baseline. Thus, BHB concentration- and time-dependently rescued LPS-induced BV2 microglial circularity, which is associated with inflammatory potency.

### BHB concentration-dependently and significantly reduced inflammatory cytokine transcript levels

**FIGURE 2:**
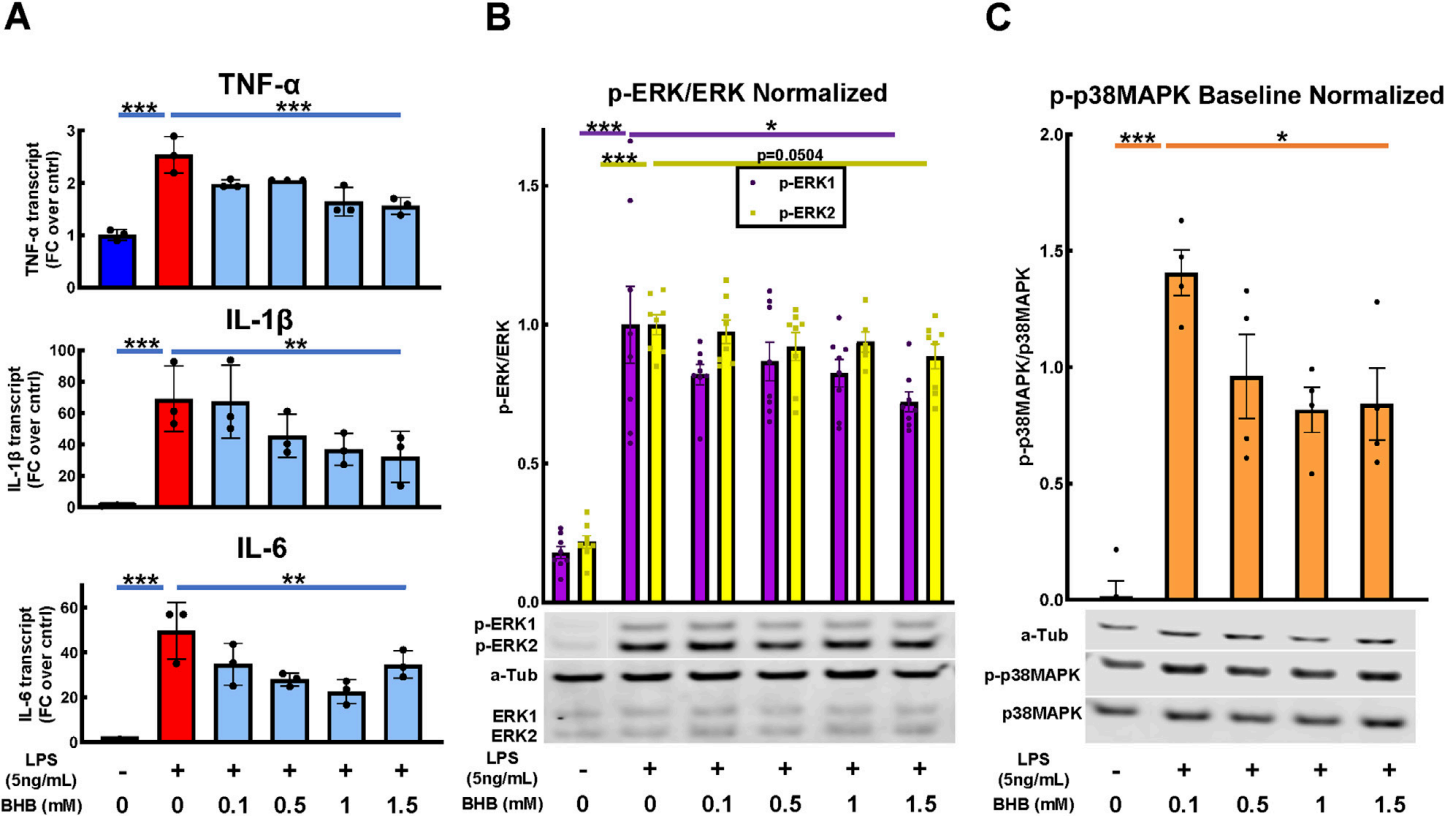
BHB concentration-dependently and significantly decreased pro-inflammatory cytokines 4 hrs after LPS was added, and upstream p-ERK and p-38MAPK signaling 1 h after LPS was added. BV2 cells were plated, incubated overnight, treated with LPS and BHB, and then incubated for a specified interval before samples were collected and processed for either qRT-PCR or Western blot. Bars are mean ± SEM. One-way ANOVA was used for a linear trend test comparing groups treated with LPS, followed by *post hoc* Dunnett’s tests for multiple comparisons, which compared baseline control and BHB exposure groups to the [LPS] control. * = p < 0.05, ** = p < 0.01, and *** = p < 0.001. **(A)** BHB concentration-dependently and significantly decreased LPS-activated transcription of canonical inflammatory cytokines IL-6, TNF-α, and IL-1β; three technical replicates per group. **(B)** BHB concentration-dependently decreased LPS-activated p-ERK1 and p-ERK2 signaling 1 h after 5 ng/mL LPS was added, and this was significant for p-ERK1. The test for p-ERK2 had p = 0.0504, with eight biological replicates per group. Representative Western blot images are shown. **(C)** BHB concentration-dependently and significantly decreased LPS-activated p-p38MAPK signaling 1 h after 5 ng/mL LPS was added. Data were baseline normalized to subtract homeostatic p38MAPK signaling, which was constitutively high in this cell type, with four biological replicates per group. Representative Western blot images are shown.

Figure 2 shows the BHB and LPS effects on inflammatory cytokine transcript levels, and upstream intracellular signaling. As expected, LPS significantly activated the transcription of canonical inflammatory cytokines: IL-6, TNF-α, and IL-1β. In contrast, BHB concentration-dependently and significantly decreased the transcription of these same cytokines, reaching a maximum of ∼50% inhibition at 1.5 mM BHB (Figure 2A).

Next, we tested upstream signaling pathways that drive the expression of these inflammatory transcripts: ERK and p38 mitogen-activated protein kinase (p38MAPK) pathways. After LPS is added, phospho-ERK1/2 (p-ERK) and phospho-p38MAPK (p-p38MAPK) significantly increase, which indicates an increase in their activity. In contrast, BHB concentration-dependently and significantly decreased p-ERK1 and p-p38MAPK signaling up to 1.5 mM BHB (Figures 2B, C), thus suggesting that BHB downregulates inflammatory pathways even in the face of LPS.

### Blocking MCT transporters or suppressing MCT expression suppressed BHB’s anti-inflammatory effects

**FIGURE 3:**
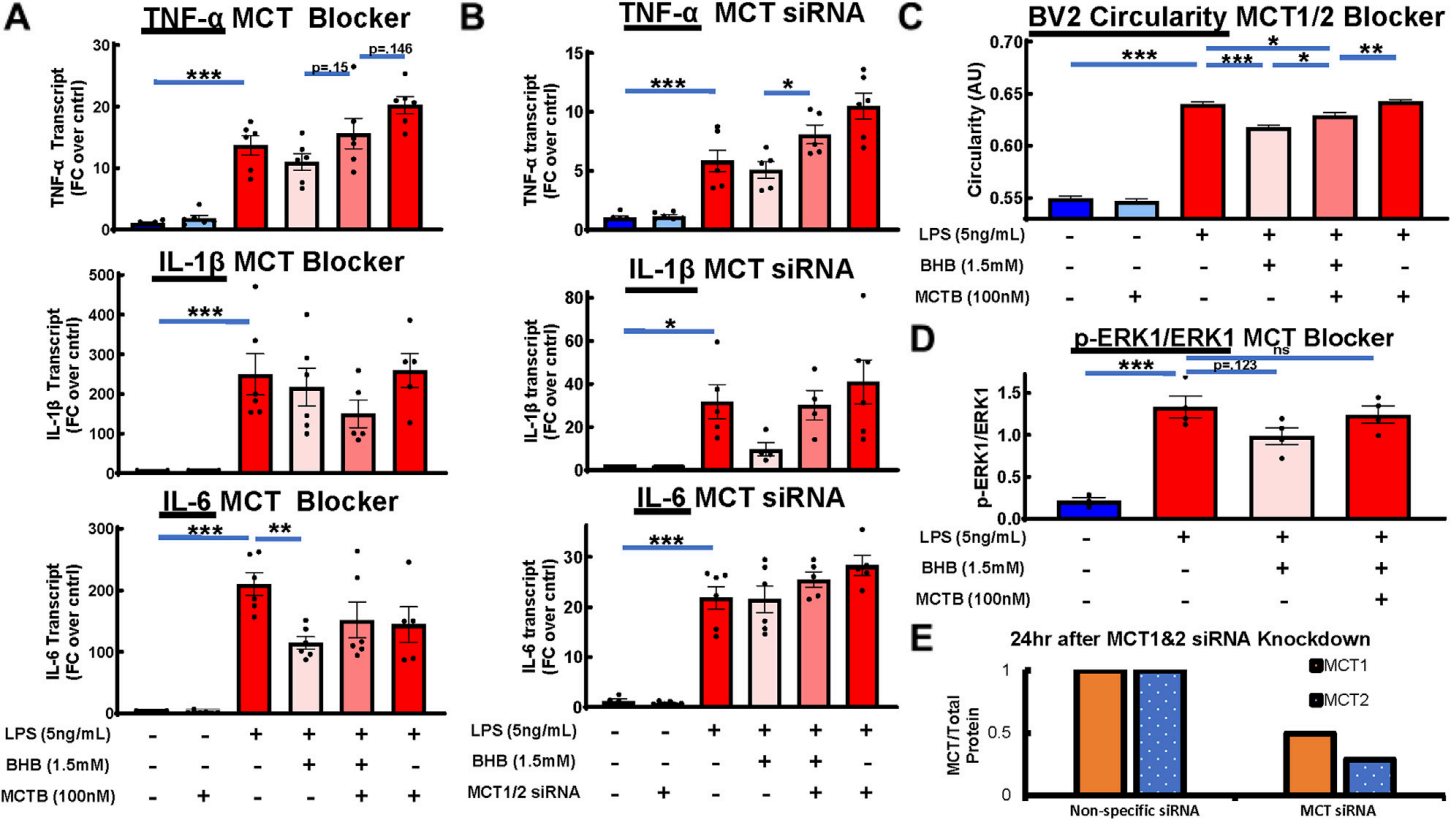
Pharmacologic and genetic MCT1/2 inhibition reversed BHB’s anti-inflammatory effect on cytokine transcripts. Pharmacologic MCT inhibition also reversed BHB’s effect on microglial circularity and p-ERK signaling. MCT1/2 are transporters that allow BHB to enter the cell. AR-C155858 (MCT blocker (MCTB)) is a potent pharmacologic MCT1 and MCT2 blocker. We used this blocker at 100 nM, which is known to inhibit 85% of MCT function. siRNA-mediated downregulation of MCT1 and MCT2 expression was also used. For all graphs: bars are mean ± SEM; one-way ANOVA with six levels was used, followed by Sidak’s *post hoc* pairwise group comparisons. Pre-determined comparisons that were tested were as follows: baseline control vs. LPS, LPS vs. LPS + BHB, LPS + BHB vs. LPS + BHB + MCTB, LPS vs. LPS + BHB + MCTB, and LPS + MCTB vs. LPS + BHB + MCTB. * = p <0.05, ** = p <0.01, and *** = p < 0.001. **(A)** MCT blocker partially inhibited BHB’s anti-inflammatory effects on transcript levels. Cells were plated, incubated overnight, and then treated with combinations of LPS, BHB, and MCT blocker. IL-6, TNF-α, and IL-1β transcriptional output were measured. Notably, the [LPS + BHB + MCTB] group and the [LPS + MCTB] group elicited different responses for TNF-α and IL-1β, suggesting that there are BHB-mediated effects that are not dependent on MCT-mediated BHB transport, with six technical replicates per group. **(B)** Genetic suppression of MCT1/2 had a similar profile as pharmacologic MCT1/2 suppression, and it partially reversed BHB’s anti-inflammatory effects on cytokine expression. BV2 cells were plated, incubated overnight, transfected with siRNA against MCT1and2, incubated overnight again, supplemented with 1.5 mM BHB and 5 ng/mL LPS, and then incubated for 4 h. Samples were collected for qRT-PCT. Knocking down MCT1/2 expression with siRNA ablated part of BHB’s anti-inflammatory effect on transcript levels. Similar to the MCTB data, the [LPS + BHB + siRNA] group and the [LPS + siRNA] group elicited different responses for all three transcripts, albeit not being significantly different, suggesting that there were BHB-mediated effects that are not dependent on MCT-mediated BHB transport, with six technical replicates per group. **(C)** Blocking BHB’s entry into the cell with pharmacologic monocarboxylate transporter blocker (MCTB) partially inhibited its anti-circular effect on microglial morphology. Cells were plated, incubated overnight, treated with combinations of MCTB, BHB, and LPS, and then assayed 8 h later. The same Cytation5 fluorescent imaging and data reduction protocol from Figure 1 was used to generate bar graphs. Blocking BHB’s entry into the cell with MCTB eliminated ∼45% of BHB’s anti-circular effect. However, like with the transcript data, MCTB did not eliminate all the anti-circular effect from BHB, suggesting that BHB had effects that are not dependent on MCT. Once we remove BHB, even with MCTB and LPS, microglial morphology returned to LPS-only levels. In this experiment, MCTB had no effect on its own on BV2 microglial morphology (∼10,000 individual cell measurements per group). **(D)** Blocking BHB’s entry into the cell with MCT blocker eliminated BHB’s downregulation of LPS-activated p-ERK signaling 1h after LPS was added. Cells were plated, incubated overnight, and then treated with LPS, LPS + BHB, or LPS + BHB + MCT blocker, and p-ERK and ERK levels were measured by Western blot, with four biological replicates per group. One-way ANOVA with four levels was used, followed by the Sidak’s *post hoc* group comparisons. Predetermined comparisons tested were as follows: baseline control vs. LPS, LPS vs. LPS + BHB, LPS + BHB vs. LPS + BHB + MCTB, and LPS vs. LPS + BHB + MCTB. * = p < 0.05, ** = p < 0.01, and *** = p < 0.001. **(E)** MCT1 and 2 protein over total protein with nonspecific and MCT siRNA were measured by Western blot, showing the degree of knockdown of MCT protein with the siRNA approach. Cells were plated, incubated overnight, transiently transfected with siRNA, and incubated for another day; then, cell pellets were collected, lysed, and ran through Western blot protocol. **(E)** siRNA knockdown of MCT1 and MCT2 reduced expression by 50%–75% 24 h after transfection, as measured by Western blot. BV2 cells were plated, incubated overnight, transfected the next afternoon, and incubated overnight again, and then the cells were lysed and ran through Western blot protocol. Bars are means (normalized to nonspecific siRNA control; n = 2).

BHB enters the cell through plasma membrane transporters monocarboxylate transporter 1 (MCT1) and monocarboxylate transporter 2 (MCT2) (Newman and Verdin, 2017), and there exists a specific inhibitor of these transporters, AR-C155858 (Ovens et al., 2010), which is hereafter referred to as monocarboxylate transporter blocker (MCT blocker or MCTB). At 30 min before adding BHB, cells were exposed to 100 nM MCT blocker, with the known 85% inhibitory concentration (Ovens et al., 2010), whereas the rest of the protocol was identical to that shown in Figure 2A. BHB’s anti-inflammatory effect on LPS-driven IL-6 and TNF-α transcript levels was reversed when MCT blocker was administered (Figure 3A). Additionally, as a confirmation test, we genetically knocked down MCT1/2 with siRNA and saw a similar trend as we did with pharmacologic inhibition (Figure 3B). Although these results were generally nonsignificant, they showed a trend that MCTB at least partially reversed the anti-inflammatory effect of BHB.

### Blocking MCT transporters also inhibited BHB’s suppression of p-ERK and circularity

An amount of 100 nM of MCT blocker was added 30 min before administering 1.5 mM BHB, whereas the rest of the protocol was identical to that shown in Figure 2B. The addition of MCTB eliminated BHB’s suppression of LPS-stimulated p-ERK signaling (Figure 3D). In addition, MCTB significantly blunted BHB’s anti-circularity morphologic effect. The significant effect of MCTB was evident when we compared the [LPS + MCTB + BHB] group to the [LPS + BHB] group (Figure 3C).

### BHB significantly suppressed triggering receptor expressed on myeloid cells 2 (TREM2) expression, which was reversible by MCT blockade

**FIGURE 4:**
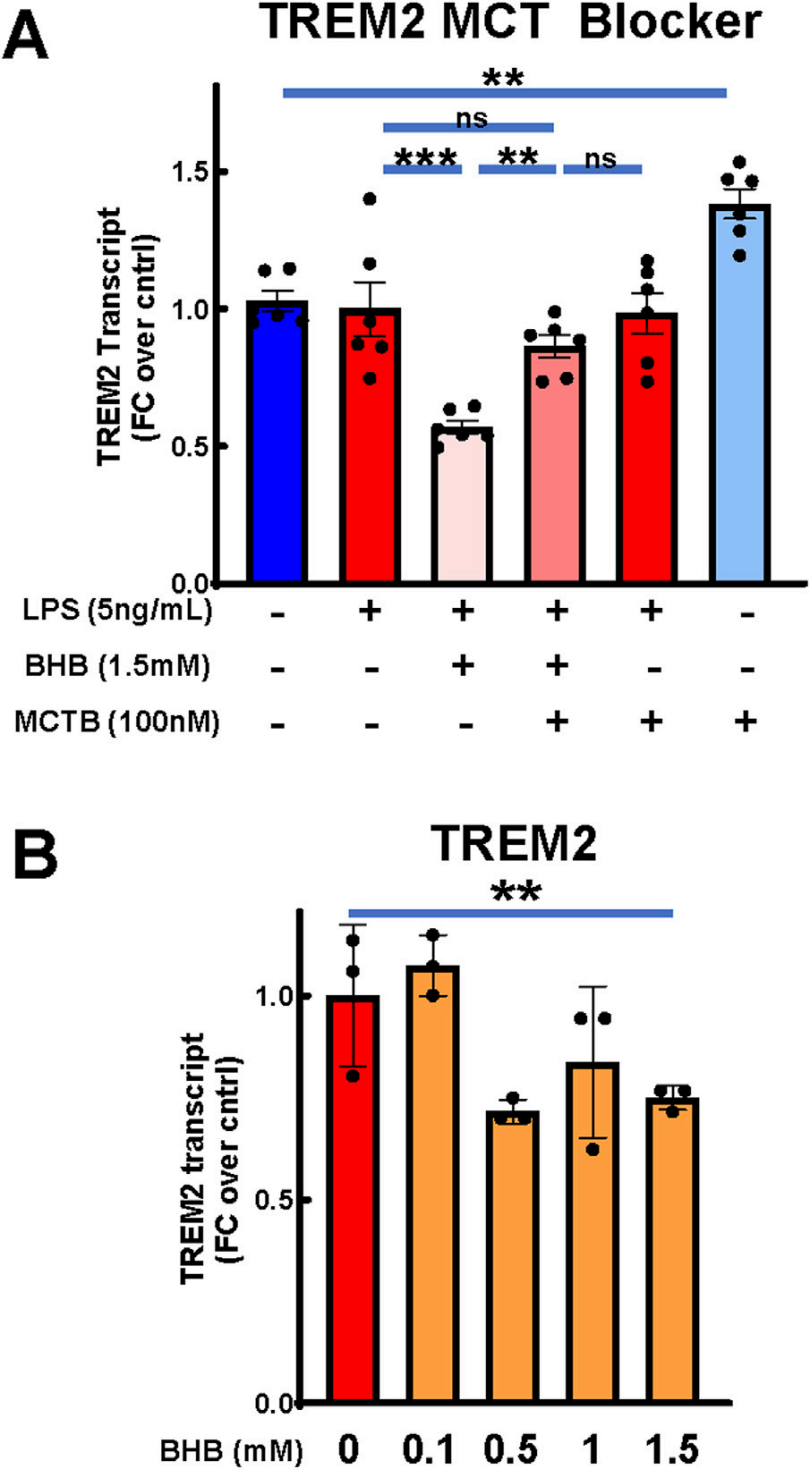
BHB significantly reduced microglial TREM2 expression, and this was significantly abrogated by MCT blocker. Cells were plated, incubated overnight, and then treated with combinations of LPS, BHB, and MCT blocker (AR-C155858), and TREM-2 transcriptional output was measured 4 h later. Bars are mean +SEM. **(A)** When treated with MCT blocker, BHB’s effect on TREM2 transcript levels in BV2 microglia was inhibited, with six technical replicates per group. One-way ANOVA with six levels was used, followed by the Sidak’s *post hoc* group comparisons. Preselected comparisons tested were as follows: baseline control vs. LPS. LPS vs. LPS + BHB, LPS + BHB vs. LPS + BHB + MCTB, LPS vs. LPS + BHB + MCTB, LPS + MCTB vs. LPS + BHB + MCTB, and baseline control vs. MCTB. * = p < 0.05, ** = p < 0.01, and *** = p < 0.001. **(B)** BHB mildly, but significantly and concentration-dependently, decreased transcription of TREM2 with 5 ng/mL LPS added to all groups, with three technical replicates per group. One-way ANOVA was used for a linear trend test. * = p < 0.05, ** = p < 0.01, and *** = p < 0.001.

Changes in TREM2 expression are connected to AD pathophysiology (Gratuze et al., 2018), and TREM2 variants confer AD risk (Benitez et al., 2013; Jin et al., 2014; Jin et al., 2015; Jonsson et al., 2013; Guerreiro et al., 2013). BHB significantly decreased TREM2 expression, and this effect appeared concentration dependent (Figure 4B). MCT blockade significantly abrogated BHB’s TREM2 effect (Figure 4A). These data support that BHB’s suppression of TREM2 required plasmalemmal BHB uptake.

### BHB increased transcription of known anti-inflammatory cytokines IL-10 and TGF-β, and CD206

**FIGURE 5:**
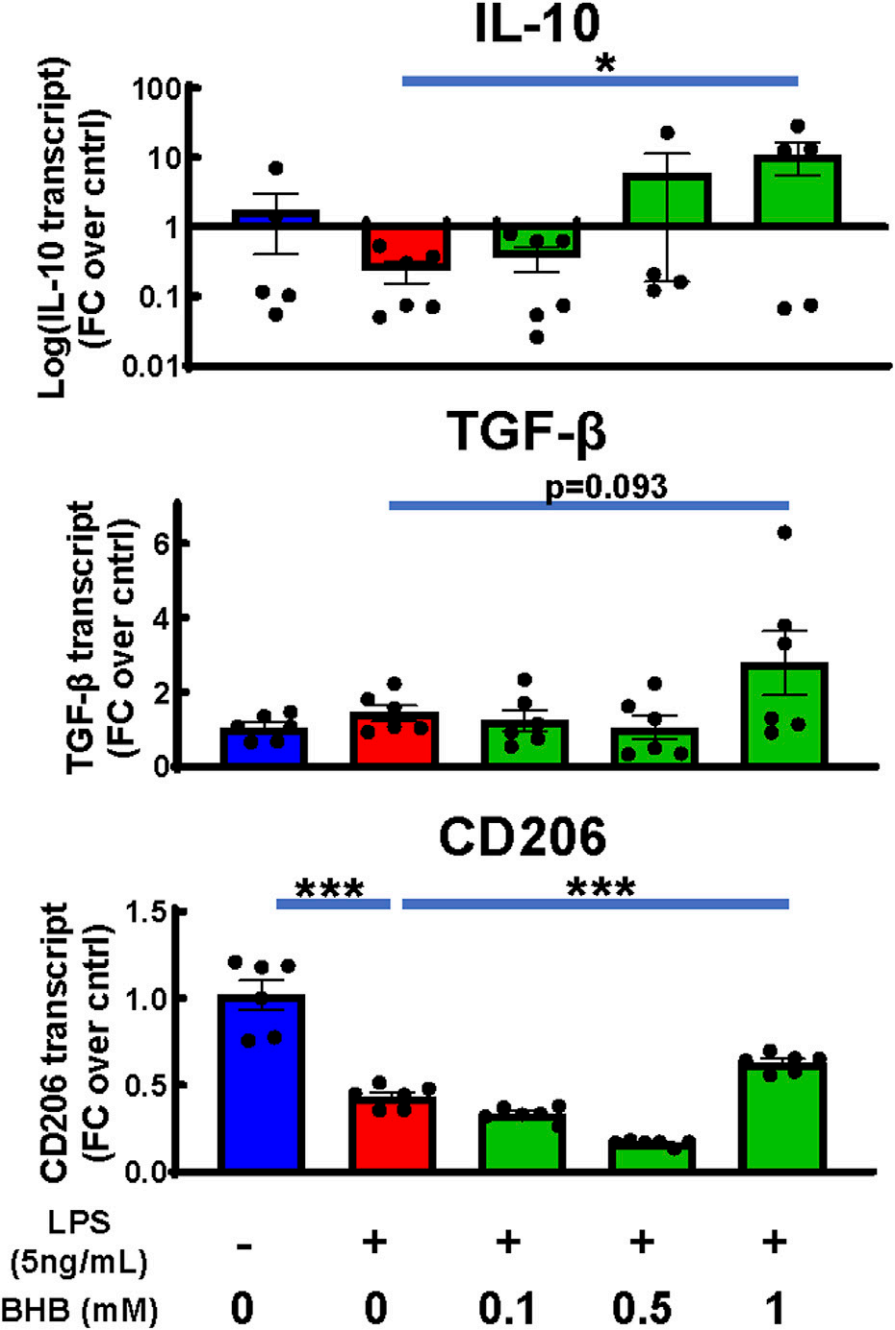
BHB rescued or increased anti-inflammatory markers under the same conditions as Figure 2A when extended to 24 hrs. Same conditions and methodology were used as for the cell experiment, RNA isolation, cDNA synthesis, and q-PCR in Figure 2A. The only difference is the incubation in BHB and LPS was extended to 24 h instead of 4 h, to measure the peak time-point for anti-inflammatory transcripts. Bars are mean ± SEM, with six technical replicates per group. One-way ANOVA was used for a linear trend test comparing groups treated with LPS, followed by *post hoc* Dunnett’s tests for multiple comparisons, which compared baseline control and BHB exposure groups to the [LPS] control. Left-skewed IL-10 data were log-transformed prior to statistical analysis. * = p < 0.05, ** = p < 0.01, and *** = p < 0.001.

Whereas the above experiments were focused on BHB’s suppression of inflammatory microglial cytokines, we also measured BHB’s effects on known *anti-inflammatory microglial* cytokines, which can be elicited on a later time course: 24 h. While LPS suppressed transcription of IL-10 and CD206, once 1 mM BHB was added, all three transcripts increased above [LPS] the control; for IL-10, this effect was concentration-dependent and significant (Figure 5). For CD206, this effect significantly varied by BHB concentration (Figure 5).

### Viability measurements via direct cell count and ATP concentration confirmed that the tested experimental conditions did not affect BV2 cell viability

We tested cell viability in response to increasing BHB concentration and confirmed via direct cell count (using Calcein AM fluorescent dye that only stains live cells) and ATP concentration that BHB does not affect cell viability (Supplementary Figure S1A). Additionally, we tested whether inhibiting MCT function influenced viability via the direct cell count method, and we observed no significant effects (Supplementary Figure S1B, C).

## Discussion

We previously showed that a KD that produced significantly higher BHB levels than a CD preserved age-related memory loss in C57BL6 mice (Roberts et al., 2017), suggesting that BHB levels themselves may be a major driver of the KD’s benefit. We subsequently showed that the KD decreased inflammatory cytokines, suggesting that BHB itself may be concentration-dependently driving its anti-inflammatory effect (Zhou et al., 2023). In addition, we found that BHB was concentration-dependently anti-inflammatory to *human* iPSC microglia (Jin et al., 2023), but that did not necessarily explain its anti-inflammatory and pro-memory effect in *mouse* model AD brains (Di Lucente et al., 2024a; Wu et al., 2020; Di Lucente et al., 2024b), so we chose a mouse microglial cell model, BV2.

The goal of this work was to establish whether or not BHB has concentration-dependent anti-inflammatory effects on mouse microglial cells, which could explain the KD’s reduction of inflammatory cytokines systemically in mice (Zhou et al., 2023), the KD’s unique memory benefit vs. CD (Roberts et al., 2017), and the KD and BHB’s anti-inflammatory and pro-memory benefit in mouse AD models PS1/APP (Jin et al., 2023) and 5XFAD (Wu et al., 2020; Di Lucente et al., 2024b). Furthermore, we aimed to better define the optimal concentration range of BHB for the anti-inflammatory effect within the physiological range of blood BHB observed *in vivo* in mice and humans on a standard CD (<0.5 mM BHB) vs. a KD (1.5 mM).

### Strengths, limitations, and suitability of the BV2 microglial cell model

BV2 microglia are a well-studied and characterized immortalized microglial cell model originally isolated from female C57Bl6 mice (Nissen, 2017a). Because of their reduced variability compared to primary microglia and their ability to be easily cultured and manipulated for experimentation, BV2 microglia are a very useful research tool. However, like all immortalized cell lines, they have their drawbacks, which must be acknowledged. Generally, the suitability of immortalized cell lines for experimentation has recently been questioned due to disparities between their behavior and that of their primary cell counterparts (Carter and Shieh, 2010). The very thing that makes them an easy-to-use research tool, the expression of oncogenes allowing quick proliferation and easy survival in culture, may significantly alter their biologic behavior compared to primary *in vivo* counterparts (Carter and Shieh, 2010; Stansley et al., 2012). Furthermore, BV2 basal morphology may differ and generally be more amoeboid than what would be seen from primary microglia *in vivo* (Horvath et al., 2008). That being said, BV2 microglia have been observed to maintain many core characteristics and functions of primary microglia, such as the expression of classic microglial cell markers, ability to respond to inflammatory stimuli, cytokine transcription, and phagocytosis (Kopec and Carroll, 1998; Blasi et al., 1990). Furthermore, and particularly relevant to our work in this study, BV-2 microglia were found to express 90% of the same genes as primary microglia in response to LPS; however, BV2’s upregulation of cytokines in response to LPS was far less pronounced than that of primary microglia (Henn et al., 2009).

### Effects of sex on microglia and BHB metabolism

Something very important to note is the effect of sex on biological behavior. This is especially important to discuss in the context of microglia and their reactivity as many neurodegenerative diseases are simultaneously linked to dysregulated microglial biology and the higher prevalence in females, including but not limited to AD (Knopman et al., 2021; Gao et al., 2023). A full review of the effect of sex on microglial biology is complex, still needs significantly more investigation, and outside the scope of this work, but what is known has been reviewed by others (Nissen, 2017b; Lynch, 2022). Although there are many complexities to the effects of sex, and many of them are life-stage-dependent, two big themes emerge: one is that adult female microglia are generally more responsive to inflammatory stimuli and have higher basal inflammatory tone; the other is that the effect of sex has both a genetic basis and a hormonal basis (Nissen, 2017b; Lynch, 2022). The X-chromosome has the highest concentration of immune-related genes throughout the entire genome, and this may explain the higher incidence of autoimmune disorders in females (Bianchi et al., 2012). Additionally, female and male microglia express estrogen receptors but preferentially different isoforms, which potentially explains the differential effects of sex (Nissen, 2017b; Lynch, 2022). It is important to note that gene-based sex effects exist, but not hormonal sex effects, in our experiments because there are no sex hormones in our cell cultures. Even less well studied is the effect of sex on BHB dynamics and metabolism, especially on the KD. In female mice on a KD, it has been shown that as they age, they are more reliant on fat metabolism than males and maintain higher plasma BHB concentrations (Smolensky et al., 2023). In theory, this may mean that females could uniquely benefit from therapeutic ketosis in the AD context, but we do not know how sex affects microglial responses to BHB. More investigation is warranted.

### LPS induces BV2 morphologic circularity that is concentration- and time-dependently suppressed by BHB at physiological BHB levels that are sustainably achieved on the KD but not on CD

Microglial function and activation state are closely tied to their morphology. *In vivo*, microglia reside in the brain and spinal cord, with long branches to sense their environment and then respond to stimuli. Once they sense injury, infection, *etc*., they retract their processes, become more circular (i.e., amoeboid) so they can move to the site of the injury/infection, produce cytokines, and otherwise respond appropriately (Huang et al., 2018; Vidal-Itriago et al., 2022). More circular microglia are associated with more reactive and inflammatory phenotypes, whereas more branched and less circular microglia are associated with less reactive/anti-inflammatory phenotypes (Vidal-Itriago et al., 2022). Furthermore, direct changes in cell morphology may directly alter the reactivity/activation state, as has been demonstrated for innate immune-cell macrophages (McWhorter et al., 2013). Thus, changes in microglial morphology are a useful biomarker of their reactivity and inflammatory potential.

As Figure 1 shows, LPS increased circularity, which is strongly correlated with inflammatory potency (Vidal-Itriago et al., 2022), and this circularity is concentration- and time-dependently suppressed by BHB. Another way of saying this is that BHB concentration- and time-dependently induces ramified, homeostatic microglial morphology within the physiological concentration range of BHB that is sustainably achieved on a KD but not on a CD. BHB decreased circularity even in the absence of LPS (Supplementary Figure S2A), suggesting that BHB’s anti-inflammatory effect is not dependent on the presence of an inflammatory stimulus. BHB’s circularity suppression was significantly blunted by MCT blockade, supporting the view that BHB plasmalemmal transport is required for circularity suppression (Figure 3C). However, we observed that BHB’s anti-circularity effects were not completely abrogated by MCT blockade. Two possible explanations occur to us: 1) BHB may be entering the cell via non-MCT-dependent mechanisms and 2) BHB may mediate anti-inflammatory effects on microglia extracellularly.

### LPS activated p-ERK and p-p38MAPK pathways and the expression of downstream inflammatory cytokines IL-6, TNF-α, and IL-1β. BHB significantly and concentration-dependently suppressed these inflammatory effects

LPS activated the phospho-ERK1/2 and phospho-p38MAPK pathways that drive microglial inflammation (Murali and Rao, 2001; Shi et al., 2002; Chen et al., 2019; Roux and Blenis, 2004; Guha et al., 2001), and BHB concentration-dependently suppressed them (Figures 2B, C). These signaling pathways control LPS-mediated transcription of canonical inflammatory cytokines IL-6, TNF-α, and IL-1β. We observed that LPS significantly upregulated the transcription of these inflammatory cytokines, whereas BHB significantly and concentration-dependently suppressed them (Figure 2A). We also tested the effect of BHB on LPS-induced cytokine secretion on human HMC3 microglia because HMC3 microglia inducibly secrete IL-6 at the LPS concentrations we investigated in contrast to BV2 cells (Dello et al., 2018). It is known that BV2 inflammatory responses and cytokine secretion are attenuated compared to primary microglia when stimulated with LPS (Henn et al., 2009). We found that BHB significantly decreased LPS-stimulated IL-6 secretion at 1 mM and 5 mM (Supplementary Figure S3), which is the concentration range of blood BHB achievable only on a KD. Furthermore, these BHB-driven anti-inflammatory effects were abrogated by MCT blockade (Figures 3A–B), the known cellular transporters for BHB, supporting the view that BHB’s MCT-mediated uptake is required for some of BHB’s microglial anti-inflammatory effects. These data support the view that BHB not only concentration-dependently suppresses *morphological* changes consistent with inflammation observed in Figure 1 but also induces significant *anti-inflammatory* changes in microglial cytokine gene expression and the underlying p-ERK and p-p38MAPK cascades that drive that inflammation (Figures 2A–C).

Taken together, these data from Figures 1–3 suggest that BHB downregulates LPS-induced inflammatory processes starting at the beginning, signal transduction (Figures 2B, C), continuing through cytokine and transcriptional-level changes (Figure 2A), and all the way to changing the whole cell morphology (Figures 1A–C), and these effects can be blocked at the beginning by blocking BHB’s cellular uptake through MCT1/2 (3A–D). From the standpoint of the microglia in the brain that receive BHB through the blood–brain barrier, this supports the view that physiological BHB levels achievable on the KD could have significant anti-inflammatory effects on brain microglia *in vivo*.

### Inhibiting BHB uptake through MCT reversed some of BHB’s anti-inflammatory effects, possibly suggesting extracellular BHB effects in these measured outputs

We believe that we are the first to address the pharmacological mechanism of BHB’s anti-inflammatory effect in BV2 microglia. We believe there are at least two, and possibly three, mechanisms that explain the overall results.

If we look at the inflammatory characteristic of circularity in the top right of Figure 3C, LPS significantly increases circularity, BHB significantly lowers it, and LPS + BHB + MCTB significantly raises circularity. This means that MCTB is reducing the anti-inflammatory potency of BHB, and as MCTB blocks BHB’s cell entry, we interpret this to mean that BHB entry is required to elicit BHB’s anti-inflammatory effect. In addition, something similar is observed in Figure 3D, where BHB reduces the central driving force of inflammation, phospho-ERK pathway activation, and it is blunted by MCTB that blocks BHB’s entry into the cell. This is mechanism 1.

Although blocking BHB’s uptake into the cell via pharmacologic or genetic means reversed part of BHB’s anti-inflammatory, anti-circular, and transcript effects, it did not reverse the whole effect (Figures 3A–C). In Figure 3A, MCTB + BHB + LPS does not *completely* bring the inflammatory endpoints back to the LPS baseline. This suggests that even when we inhibited BHB’s cellular uptake, BHB still had an anti-inflammatory effect. We saw this same trend in the MCT siRNA and MCTB circularity data; however, it is not significant (Figures 3B, C). Thus, this suggests that BHB either gained access to the cell through incomplete MCT inhibition, MCT-independent mechanisms, and/or it exerted some of its anti-inflammatory effect through not only intracellular pathways but also extracellular pathways, namely, hydrocarboxylic acid receptor 2 (HCA2) (Gonzatti and Goldberg, 2024), that is, mechanism 2; HCA2, also known as GPR109A, is a cell-surface GPCR that can bind niacin, BHB, and other monocarboxylic acids. HCA2 signaling has been documented to be anti-inflammatory in multiple studies in macrophages/microglia (Rahman et al., 2014; Offermanns and Schwaninger, 2015; Graff et al., 2016). In addition, knockdown of HCA2 via siRNA does significantly rescue the suppression of circularity induced by BHB (Supplementary Figure S4A). We interpret these data to mean that BHB elicits its anti-inflammatory effects in two ways: 1) through cell entry, which results in the activation of the p-ERK pathway that can be blocked with MCTB, and 2) through extracellular engagement of HCA2.

We also observed a trend from the MCTB and MCT siRNA transcript data that the [LPS + MCTB] group often had greater transcript expression than the [LPS] group alone. However, loss of MCT and its function alone had no significant effect on inflammatory transcript expression. Our third hypothesis is that there are likely other monocarboxylates *in addition to BHB* in the cell culture media that are anti-inflammatory. When these additional, anti-inflammatory monocarboxylates are prevented from entering the cell by MCTB, LPS could drive the inflammatory potential higher (mechanism 3). However, it is beyond the scope of this manuscript to isolate other, novel anti-inflammatory monocarboxylates; our goal is to better understand the anti-inflammatory mechanism of BHB. Despite lack of statistical significance, these multiple independent experiments in aggregate paint the same story that blocking BHB’s entry into the cell via either pharmacologic or genetic means at least partially, if not fully, reverses BHB’s anti-inflammatory effects.

### Differences between MCT blocker and siRNA data may be due to differences in the experimental approach

While the trend was the same in the inflammatory cytokine transcript data between the MCTB and siRNA experiments, in the siRNA experiment, the anti-inflammatory effect of BHB and the reversal of this effect was not as pronounced as that seen in Figures 2A, 3A. We have two hypotheses as to why: 1) there were slightly different experimental conditions between the two approaches because siRNA knockdown of MCT1/2 added 24 h to the experimental timeline. These BV2 cells double every 12 h. Thus, there were ∼4x more cells at the time of experiment for siRNA experiments vs. MCTB experiments, and cell confluency can significantly change metabolic transcriptional profiles of cells in culture, including BHB. 2) The amount of protein knockdown achieved, which was ∼50–75% (Figure 3E), in contrast to the 85% inhibition of MCT1/2 via MCT blocker (Ovens et al., 2010) is another potential cause for the discrepancy. Thus, we see the same trend of results with the MCT blocker experiment (Figure 3A) and siRNA MCT knock down, and we hypothesize that the mild differences are due to experimental limitations with the siRNA technique.

### Anti-inflammatory effects we observed are similar to the published reports of BHB’s effects in related lineage macrophages

Although reports of the effects of BHB in microglia are limited, there is more published research of BHB’s effects in functionally and developmentally similar cell types: monocytes and macrophages. In one study, BHB inhibited the activation of monocyte/macrophage NLRP3 inflammasome activation, which is a driver of some inflammatory diseases (Youm et al., 2015). In another study, BHB also ameliorated colitis by pushing macrophages toward an anti-inflammatory phenotype (Huang et al., 2022). BHB also induced a neuroprotective phenotype in monocytes/macrophages in an ischemic brain injury study (Rahman et al., 2014). These published reports in conjunction with our data support the hypothesis that BHB has anti-inflammatory effects in macrophage-like innate immune cells throughout the body.

### BHB downregulated TREM2 expression, and this was dependent on BHB’s cellular uptake

We observed that BHB downregulated TREM2 expression in this BV2 microglial *in vitro* system (Figure 4B), and this effect was dependent on BHB’s uptake through MCT1/2 (Figure 4A). TREM2 is a microglial cell surface receptor that senses multiple lipid carriers, including ApoE4, HDL, and LDL status (Damisah et al., 2020; Nugent et al., 2020; Li et al., 2022), and it is intimately linked to microglial metabolism (Zheng et al., 2018). Microglia express TREM2, and its signaling promotes core, AD-relevant physiological functions of microglia: phagocytosis, chemotaxis, survival, and proliferation, and physiological synaptic pruning (Hansen et al., 2018; Jay et al., 2017).

**FIGURE 6:**
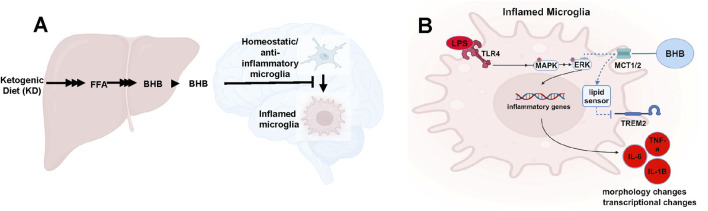
BHB is synthesized in the liver, then travels to the brain, where it antagonizes inflammatory microglial phenotypes and promotes resting/anti-inflammatory phenotypes **(A)**, and the intracellular pathway implicated in this effect **(B)**.

Loss-of-function variants in TREM2 increase AD risk (Benitez et al., 2013; Jin et al., 2014; Jin et al., 2015; Jonsson et al., 2013; Guerreiro et al., 2013). One explanatory hypothesis is that the loss of microglial lipid-sensing ability is pro-inflammatory and part of the AD pathophysiology. We observed that BHB levels sustainably achieved on KD suppressed TREM2 receptor expression. Our interpretation and testable hypothesis is that higher KD-driven BHB concentration signals a lipid-replete state, decreasing the need for TREM2-based lipid sensing and lipid import (Figure 6B). The [MCTB] alone group compared to the control in Figure 4A lends itself to this theory too but displays the other side of this paradigm: when we inhibited the transport of oxidized lipids through MCT, TREM2 expression increased, possibly because it sensed a lipid-depleted state.

### BHB increases microglial anti-inflammatory, homeostatic transcripts

BHB increased anti-inflammatory transcripts at 24 h after LPS/BHB was added (Figure 5). These data support the view that long-term physiological KD or BHB dosing may induce a longer-term change toward the anti-inflammatory microglial tone.

### How this work fits with the existing literature

We have already complemented these BV2 data with human iPSC data; we previously showed that BHB dose-dependently suppressed inflammation in human iPSC microglia (Jin et al., 2023). That study was much less detailed, and many more inflammatory endpoints that were absent in the previous study were investigated in this study. The previous study did not include as many cytokines (two vs. the four in this study), did not include the morphometric estimates of inflammation-dependent circularity, and did not include measurements of p-ERK and p-38MAP kinase inflammatory pathways, and TREM2. Importantly, the previous study also did not show that the anti-inflammatory effects of BHB required cellular BHB import by chemical MCT knockdown and siRNA of MCT. Thus, by using BV2 cells, we were able to address many more inflammation mechanistic endpoints and pharmacological mechanisms than the previous work, and we identified that BHB dose-dependently suppresses the p-ERK/MAPK pathway that drives the inflammation. The fact that something similar happens in human iPSC microglia confirms that what we are seeing in mouse BV2 cells is more likely to be relevant to the human physiological condition. The homology of the mouse BV2 cell results are helping in building a mechanistic understanding of this effect; that is, that in some way, BHB is dose-dependently inhibiting the central drivers of inflammation, p-ERK and p-38MAPK, but how BHB is doing it is still to be discovered.

Regarding *in vivo* AD models, we have published that BHB dosed as KD or BHB itself reverses memory deficits in two *in vivo* mouse models of AD, PS1/APP (Di Lucente et al., 2024a) and 5XFAD (Di Lucente et al., 2024b), and our preliminary data in ApoE4 mice show something similar. Furthermore, other groups have shown similar benefit of KD/BHB in *in vivo* AD and aging mouse models (Zhou et al., 2023; Roberts et al., 2017; Pathak et al., 2022; Newman et al., 2017; Wu et al., 2020; Xu et al., 2022). There is evidence this is relevant to humans too (Phillips et al., 2021; Dyńka et al., 2022; Oliveira et al., 2024). BHB’s and KD’s potency to ameliorate multiple independent mouse models of AD is what motivated us to study BHB’s anti-inflammatory mechanism in more detail in BV2 cells.

### Summary and prospects

In mice and humans, physiological levels of ∼1.5 mM BHB are sustainably achieved uniquely on the KD but not on an *ad libitum* or one-meal-a-day CD (Zhou et al., 2023; Di Lucente et al., 2024a; Roberts et al., 2017; Pathak et al., 2022). We and others uniquely observed memory benefits in C57BL6 mice on a KD but not on the CD. In addition, in PS1/APP and 5XFAD mice, we have observed significant increases in synaptic plasticity using BHB alone (Di Lucente et al., 2024a; Di Lucente et al., 2024b). In parallel to the above memory benefits of BHB, in this work, we observed concentration-dependent anti-inflammatory effects of BHB on mouse microglial cells precisely in the concentration range of BHB that produced the memory benefit in aging C57Bl6 mice, PS1/APP and 5XFAD mice. We showed that BHB’s suppression of the p-ERK and p-MAPK pathways, inflammatory cytokine elicitation, and inflammatory morphology are all reversible by blocking BHB’s entry into the cell using an MCT inhibitor. These data support the idea that BHB, through its concentration-dependent suppression of inflammatory processes, may be driving the KD’s memory benefit, and that maximizing BHB levels through a KD or with BHB supplements could potentially support memory deficits in humans in the aging or AD context.

## Methods

For all experiments, BV2 (RRID: CVCL_0182) microglial cells (originally derived from female C57/Bl6 mice (Nissen, 2017a)) were used. They were acquired directly from AcceGen with appropriate documentation confirming their authenticity and the known genetic mutations to this cell line, which is detailed in the materials table. Cells were continuously cultured in ∼35 mL 10% fetal bovine serum (FBS) Dulbecco’s modified Eagle medium F12 (DMEM/F12) in T175 flasks. Then, they were sub-cultivated at a 1:20 (0.5 mL from 10 mL cell suspension) ratio every 2 or 3 days. Cells between passage numbers 6 to 15 were used in this study. For all plate-based assays, plate effects were not observed nor measured. To further reduce the risk of plate effects, all wells were plated, but edge wells were excluded from analysis. Whenever LPS was used, it was always at 5 ng/mL final and added at 100 x concentration. Catalog numbers and manufacturers of reagents used are summarized in Table 1.

**TABLE 1:** Materials.

Catalog Number	Manufacturer	Description
ABC-TC212S	AcceGen	BV2 murine microglial cells: C57Bl/6 origin “cells express the nuclear v-myc and the cytoplasmic v-raf oncogene products, as well as the env gp70 antigen at the surface level.” RRID: CVCL_0182
159910	Thermo Scientific	Nunc™ EasYFlask™ Cell Culture Flasks T175 flasks
156367	Thermo Scientific	Nunc™ EasYFlask™ Cell Culture Flasks T25 flasks
3603	Corning	Corning® 96-well Flat Clear Bottom Black Polystyrene TC-treated Microplates
3516	Corning	Costar™ 6-well Clear TC-treated Multiple-Well Plates, individually wrapped and sterile
G7571	Promega	Cell-Titer Glo
12500-062	Gibco	DMEM/F12
30-2003	ATCC	Eagle’s minimum essential medium (EMEM)
35010CV	Corning	Fetal bovine serum (FBS); lot 35010159
54965-10G-F	Millipore-Sigma	Beta-hydroxybutyrate (BHB)
L7895	Sigma-Aldrich	Lipopolysaccharide (LPS)
74034	Qiagen	RNeasy Plus Micro Kit
18080051	Invitrogen	SuperScript™ III First-Strand Synthesis System
A25742	Applied Biosystems	SYBR Green qPCR Master-Mix
9803S	Cell Signaling	Cell Signaling Lysis Buffer
04693116001	Roche	Complete Mini Protease Inhibitor
4906845001	Roche	PhosSTOP Phosphatase Inhibitor
WG1403BOX	Invitrogen	NuPAGE™ Bis-Tris Midi Protein Gels, 4%–12%, 1.0 mm
4377	Cell Signaling	p-ERK1/2 antibody (197G2); RRID: AB_331775
4696	Cell Signaling	ERK1/2 antibody (L34F12); RRID: AB_390780
9216	Cell Signaling	p-p38MAPK antibody (28B10); RRID: AB_331296
9212	Cell Signaling	p38MAPK antibody; RRID: AB_330713
T9026	Sigma-Aldrich	α-Tubulin antibody (DM1A); RRID: AB_477593
92632210	Licor	Goat anti-mouse IRDye 800CW Licor secondary antibody
92668072	Licor	Donkey anti-mouse IRDye 680RD Licor secondary antibody
92632211	Licor	Goat anti-rabbit IRDye 800CW Licor secondary antibody
92668073	Licor	Donkey anti-rabbit IRDye 680RD Licor secondary antibody
927-60001	Licor	Intercept TBS Blocking Buffer
HY-13248	Med Chem Express	AR-C155858
GL00250	Oz Biosciences	Glial-Mag Magnetofection Kit
4390771 (sirRNA ID: s73851)	Life Technologies	Ambion Silencer Select siRNA MCT1 Fwd: GGUCUUUCUUAGUAGUUAUtt Rev: AUAACUACUAAGAAAGACCaa
4390771 (sirRNA ID: s73858)	Life Technologies	Ambion Silencer Select siRNA MCT2 Fwd: GCAACAGCGUGAUAGAGCUtt Rev: AGCUCUAUCACGCUGUUGCtg
4390771 (siRNA ID: s96087)	Life Technologies	Ambion Silencer Select siRNA HCA2
4390843	Life Technologies	Ambion Silencer Select siRNA nonspecific
31985-070	Gibco	Opti-MEM
85680	Cell Signaling	MCT1 antibody
PA576603	Invitrogen	MCT2 antibody
80011-3	Biotium	Calcein AM
62249	Thermo Scientific	Hoechst 33342
14040-133	Gibco	Dulbecco’s phosphate-buffered saline + Ca2+ +Mg2+
Ab178013	Abcam	Human IL-6 SimpleStep ELISA Kit

Primer Set	Manufacturer	Sequence
IL-6	Integrated DNA Technologies	Fwd: AGACTTCCATCCAGTTGCCTTCT Rev: CTGTTGGGAGTGGTATCCTCTGT
TNF-α	Integrated DNA Technologies	Fwd: TGGGTTGTACCTTGTCTACTCCC Rev: GAGGAGGTTGACTTTCTCCTGGT
IL-1β	Integrated DNA Technologies	Fwd: GTGGATCCCAAGCAATACCCAAA Rev: GTGAGGTGCTGATGTACCAGTTG
TREM2	Integrated DNA Technologies	Fwd: ACTTATGACGCCTTGAAGCACTG Rev: CTTCAGAGTGATGGTGACGGTTC
β-actin	Integrated DNA Technologies	Fwd: TTACTGCTCTGGCTCCTAGC Rev: CCTGCTTGCTGATCCACATC
IL-10	Integrated DNA Technologies	Fwd: TATGCTGCCTGCTCTTACTGACT Rev: TCTAGGAGCATGTGGCTCTGG
TGF-β	Integrated DNA Technologies	Fwd: GATCCTGTCCAAACTAAGGCTCG Rev: TTAGCATAGTAGTCCGCTTCGGG
CD206	Integrated DNA Technologies	Fwd: GAAATTCCGCTGGGTGTCAGATT Rev: TGTGCCCTTGATTCCAAAGAGTG

### Cell viability assays

BV2 microglia were plated at 6e3 cells/cm^2^ in 100 µL/well with 2% FBS Dulbecco’s modified Eagle medium (DMEM) in a 96-well plate, incubated overnight, and then treated with BHB ranging from 0 to 5 mM for 20 h. Then, cell viability was assessed via two different methods: direct cell count and ATP concentration. Direct cell count was carried out by measuring cells co-stained with Hoechst 33342 and Calcein AM, which only stains live cells. ATP measurements were done using the well-validated Cell-Titer Glo method using the manufacturer protocol (Riss et al., 2024). Additionally, the viability of BV2 microglia treated with LPS, BHB, and MCTB was assessed. Cells were plated at 6e3 cells/cm^2^ in 100 µL/well 2% FBS DMEM in a 96-well plate, incubated overnight, and then treated with MCTB, LPS, and BHB for 20 h. Direct cell count was carried out by measuring cells co-stained with Hoechst 33342 and Calcein AM, which only stains live cells. Measurements are displayed as fold change compared to control.

### Morphometric analysis of microglia

Cells were plated at 6e3 cells/cm^2^ in a tissue-culture-treated 96-well plate in 100 µL/well 2% FBS Eagle’s minimal essential media (EMEM) and incubated overnight in a 37 ℃ 5% CO_2_ incubator. Each experimental group was a combination of individual cell measurements across 4–12 wells. Again, plate effects were not observed nor measured; thus, we had no *a priori* expectation of difference between identically treated wells. To further reduce the risk of plate effects, all wells were plated, but the edge wells were not analyzed. Next morning, cells were treated with 0–5 mM BHB. Cells were incubated for an additional 30 min. Cells were then treated with 0 or 5 ng/mL LPS. Cells were incubated for an additional 8 or 14 h, and 80% of cell media was removed and replaced with 1 µM Calcein AM and 4 µM Hoechst 33342 in Dulbecco’s phosphate-buffered saline (DPBS) +Ca2+ +Mg2+. This allowed for clear visualization of the cell and its boundaries (Calcein AM–GFP channel) and nuclei (Hoechst 33342–DAPI channel). Live cells were imaged at 10x in our BioTek Cytation 5 high-throughput non-confocal imager on GFP, DAPI, phase contrast, and brightfield channels. We generated a protocol in Cytation 5 Gen 5 software application to automatically generate regions of interest (ROIs) based on unmodified thresholded Calcein AM fluorescent cell images. Calcein AM only stains live cells; thus, these morphometric analyses do not reflect changes in cell viability. This system is ideal because there is no human observer bias, and the thresholding is consistent across the whole experiment. From these ROIs, we generated a circularity metric per cell, which is a metric that is on a 0–1 scale and calculated according to the formula: circularity = 4π*A/perimeter^2^. Individual cell measurements were pooled across identically treated wells. These findings were validated via a blinded observer that hand-drew cell outlines. ImageJ was used to outline cells, and from these outlines, we could measure the perimeter of the outline and area contained within to calculate circularity.

### RNA isolation, cDNA synthesis, and q-PCR

Cells were plated at 6e3 cells/cm^2^ in 5 mL 2% FBS EMEM in T25 flasks and incubated overnight in a 37°C 5% CO_2_ incubator. On the next day, cells were then switched to 5 mL 2% FBS no-glucose DMEM supplemented with 0–1.5 mM BHB. After incubating for an additional 30 min, cells were then treated with 5 ng/mL LPS. Cells were incubated for an additional four (IL-6, TNF-α, IL-1β, and TREM2 transcripts) or 24 (IL-10, TGF-β, and CD206) hours. Cells were then lysed, and RNA was isolated using a Qiagen RNeasy micro kit [RNA] and normalized. cDNA was synthesized using the SuperScriptIII First Strand Synthesis System. qPCR was run with the Powerup SYBR Green qPCR Master-Mix according to protocol: 50 °C for 2min, 94 °C for 5 min, and 38 cycle x (94 °C for 15”, 58°C for 30”). Primer sequences are shown in Table 1 in Materials section. All primers were developed against coding sequences of the corresponding protein. Data were normalized to β-actin as the housekeeping gene via the delta delta-Ct method and then to the baseline control group. β-actin was confirmed to not change across the experimental groups.

### Western blot for p-ERK1/2 p-p38MAPK signaling experiments

Cells were plated at 6e3 cells/cm^2^ in 2 mL 2% FBS EMEM in a tissue-culture-treated, 6-well plate and incubated overnight in a 37°C 5% CO_2_ incubator. On the next day, cells were then switched to 2 mL 2% FBS no-glucose DMEM supplemented with 0–1.5 mM BHB. After incubating for an additional 30 min, cells were then treated with 5 ng/mL LPS. Cells were incubated for an additional 1 h. Cells were lysed with 0.2 mL Cell Signaling lysis buffer supplemented with Roche protease and phosphatase inhibitors. Cell lysates were collected, and protein concentration was quantified using the Bradford assay with subsequent dilution with lysis buffer. An amount of 12µg protein per well (10µL/well) were run on 26-well gel NuPAGE™ Bis-Tris Midi Protein Gels. Gel electrophoresis was run at 80 V for 20 min and then at 120 V for 2 h. Protein was transferred onto 0.2 um nitrocellulose. Blots were blocked in Licor TBS blocking buffer. Blots were probed for p-ERK1/2 (1:1000), ERK1/2 (1:1000), p-p38MAPK (1:2000), p38MAPK (1:1000), and α-Tubulin (1:5000) in Licor TBS blocking buffer overnight at 4°C. Blots were washed 3 x, for 5 min each in TBST (Tween 0.1%). Licor secondary antibodies were probed at 1:15,000 in TBS blocking buffer. Blots were washed 3 x, for 5 min each in TBST (Tween 0.1%). Images were taken with Licor Odyssey CLx and analyzed with Image Studio for densitometry quantification. Boxes for quantification were uniform in size across an individual experiment. Antibodies are commercially available from Cell Signaling, whose website provides citations (hundreds if not thousands of citations per antibody because these antibodies are so widely used) and in-house validation experiments for each antibody.

### AR-C155858 MCT1/2 blocker experiments

AR-C155858 (MCTB) is a potent MCT1 and MCT2 inhibitor (Ovens et al., 2010). MCT1/2 are transporters that allow BHB to enter the cell (Newman and Verdin, 2017). For these experiments, we used 100 nM AR-C155858 because this is the lowest concentration to achieve maximal MCT1/2 inhibition, which is ∼85% transport reduction (Ovens et al., 2010). Utilizing this inhibitor required minimal alterations to the existing methods and protocols for analogous experiments (e.g., Figures 2A vs. 3A). AR-C155858 was dissolved in DMSO, and the final concentration of DMSO was 0.01%. An amount of 0.01% DMSO was maintained across all experimental groups in experiments involving AR-C155858. The inhibitor was added 30 min before dosing with BHB. All other experimental conditions were maintained the same.

### MCT1/2 siRNA knockdown

To knock down MCT1/2, we used the Oz Biosciences Glial-Mag magnetofection kit due to its reported increased transfection efficiency over other siRNA transfection protocols, lack of toxicity and inflammatory activation, design, which is specifically for microglia (reported in BV2 cells too), and ease of use (Smolders et al., 2018; Carrillo-Jimenez et al., 2018). Cells were plated at 6e3 cells/cm^2^ in 2 mL 2% FBS EMEM in a 6-well plate and incubated overnight in a 37 °C 5% CO_2_ incubator. On the next day, 2 h before transfection, cell media were switched to 2 mL 0% FBS Opti-MEM. Cells were transfected according to the manufacturer’s protocol at a ratio of 6 uL Glial-Mag transfection reagent to 1.5 ug siRNA per well in 2 mL of media in a 6-well dish. These ratios were chosen based on experimental optimization for maximal siRNA transfection efficiency. After transfection, the cells were transferred back into a 37 °C 5% CO_2_ incubator. Cells were incubated on a magnetic plate for 20 min. Cells were then removed from the plate and allowed to be incubated for an additional 2 h. Cell media were then replaced with 2 mL 1% FBS Opti-MEM. Cells were incubated overnight. Next day, cells went through the same experimental methods described in the “RNA isolation, cDNA synthesis, and q-PCR” section.

### IL-6 secretion ELISA of HMC3 cell culture supernatants

BV2 cells do not inducibly secrete inflammatory cytokines to detectable levels at our tested LPS concentrations. To test the impact of BHB on microglial inflammatory secretory function, we used HMC3s, a human microglial cell line, as a model system because of HMC3’s inducibility to secrete IL-6 (Dello et al., 2018), an important and classic inflammatory cytokine, at the concentrations of LPS that we tested. HMC3 cells were plated at 6e3 cells/cm^2 in 200 uL 10% FBS DMEM/F12 media in 96-well dishes and incubated overnight; then, the media were changed to 2% FBS EMEM and supplemented with varying concentrations of LPS (0–6.25 ng/mL) and BHB (0–5 mM) and incubated overnight again; and then, IL-6 in the media was measured the next day using Abcam IL-6 ELISA kit. Cell number was counted at the end of the experiment using Hoechst 33342 dye and the Cytation5 imager direct cell count method. Data were normalized to the cell number.

### Statistics

All statistical analyses were carried out as directed by biostatistician Dr. Kyoungmi Kim, and Dr. Kim approved the final version of all the figures and manuscript. All statistical analyses were performed using GraphPad Prism 10.2.3 (GraphPad Software Inc., San Diego, CA). Prior to statistical inference tests, the analysis of residuals was performed to validate the normality and homoscedasticity assumptions. For group comparisons, one-way ANOVA or two-way ANOVA was used, followed by Dunnett, Sidak, or Tukey’s *post hoc* tests for pairwise group comparisons with adjustment for multiple comparisons, as appropriate. For experiments testing the concentration dependence of BHB for specified outcomes, one-way ANOVA was used to test for a linear trend in the group means as a function of the concentration level, followed by *post hoc* Dunnett’s tests for comparing the BHB exposure groups to the [LPS] control group, with adjustment for multiple comparisons. Data are presented as mean ± standard error of the mean (SEM). Group sizes are noted in figure legends. The use of either biological or technical replicates is specified in the figure legends. Biological replicates are biologically distinct samples treated in the same conditions (e.g., wells), whereas technical replicates are repeated measurements from the same sample. * = p < 0.05, ** = p < 0.01, and *** = p < 0.001. Adjusted p-values for multiple testing are reported in Table 2 and figures.

**TABLE 2:** Comparisons and p-values.

Figure	Comparison	Adjusted p-values
1A	LPS + 0 mM BHB – LPS +1.5 mM BHB	<0.0001
1A	Control vs. LPS	<0.0001
1A	LPS vs. LPS +0.63 mM BHB	>0.9999
1A	LPS vs. LPS +1.25 mM BHB	0.0002
1A	LPS vs. LPS +2.5 mM BHB	0.0005
1A	LPS vs. LPS + 5 mM BHB	<0.0001
1B	LPS + 0 mM BHB – LPS +1.5 mM BHB	<0.0001
1B	Control vs. LPS	<0.0001
1B	LPS vs. LPS +0.63 mM BHB	0.9969
1B	LPS vs. LPS +1.25 mM BHB	>0.9999
1B	LPS vs. LPS +2.5 mM BHB	0.0001
1B	LPS vs. LPS + 5 mM BHB	<0.0001
2A	TNF-α: LPS + 0 mM BHB – LPS +1.5 mM BHB	0.0002
2A	TNF-a: control vs. LPS	<0.0001
2A	IL-1β: LPS+0 mM BHB – LPS+1.5 mM BHB	0.0086
2A	IL-1β: control vs. LPS	0.0009
2A	IL-6: LPS+0 mM BHB – LPS+1.5 mM BHB	0.0027
2A	Il-6: control vs. LPS	<0.0001
2B	p-ERK1/ERK1: LPS+0 mM BHB – LPS+1.5 mM BHB	0.0281
2B	p-ERK1/ERK1: control vs. LPS	<0.0001
2B	p-ERK2/ERK2: LPS+0 mM BHB – LPS+1.5 mM BHB	0.0504
2B	p-ERK2/ERK2: control vs. LPS	<0.0001
2C	p-p38MAPK/p38MAPK: LPS+0.1 mM BHB – LPS+1.5 mM BHB	0.0112
2C	p-p38MAPK/p38MAPK: control vs. LPS +0.1 mM BHB	<0.0001
3A	TNF-α: control vs. +LPS	<0.0001
3A	TNF-α: +LPS vs. +LPS + BHB	0.6676
3A	TNF-α: +LPS + BHB + A vs. +LPS + BHB	0.1506
3A	TNF-α: +LPS vs. +LPS + BHB + A	0.8924
3A	TNF-α: +LPS + MCTB vs. +LPS + BHB + A	0.1459
3A	IL-1β: +LPS + BHB vs. +LPS	0.9770
3A	IL-1β: +LPS + BHB + AR vs. +LPS + BHB	0.6805
3A	IL-1β: +LPS + BHB + AR vs. +LPS	0.2924
3A	IL-1β: +LPS + MCTB vs. +LPS + BHB + AR	0.2490
3A	IL-1β: control vs. +LPS	0.0001
3A	IL-6: control vs. +LPS	<0.0001
3A	IL-6: +LPS vs. +LPS + BHB	0.0046
3A	IL-6: +LPS + BHB + AR vs. +LPS + BHB	0.5860
3A	IL-6: +LPS + BHB + AR vs. +LPS	0.1473
3A	IL-6: +LPS + MCTB vs. +LPS	0.1037
3B	IL-1β: +LPS + BHB vs. +LPS	0.1686
3B	IL-1β: +LPS + BHB + siRNA vs. +LPS + BHB	0.2810
3B	IL-1β: +LPS + BHB + siRNA vs. +LPS	>0.9999
3B	IL-1β: +LPS + siRNA vs. +LPS + BHB + siRNA	0.7883
3B	IL-1β: control vs. +LPS	0.0155
3B	TNF-α: +LPS + BHB vs. +LPS	0.9616
3B	TNF-α: +LPS + BHB + siRNA vs. +LPS + BHB	0.0431
3B	TNF-α: +LPS + BHB + siRNA vs. +LPS	0.1940
3B	TNF-α: +LPS + siRNA vs. +LPS + BHB + siRNA	0.1434
3B	TNF-α: control vs. +LPS	0.0003
3B	IL-6: +LPS + BHB vs. +LPS	>0.9999
3B	IL-6: +LPS + BHB + siRNA vs. +LPS + BHB	0.5479
3B	IL-6: +LPS + BHB + siRNA vs. +LPS	0.6152
3B	IL-6: +LPS + siRNA vs. +LPS + BHB + siRNA	0.8428
3B	IL-6: control vs. +LPS	<0.0001
3C	-AR + BHB + LPS vs. -AR -BHB + LPS	<0.0001
3C	+AR + BHB + LPS vs. -AR + BHB + LPS	0.0223
3C	+AR + BHB + LPS vs. -AR -BHB + LPS	0.0287
3C	+AR - BHB + LPS vs. +AR + BHB + LPS	0.0041
3C	-AR - BHB - LPS vs. - AR - BHB + LPS	<0.0001
3D	+LPS + BHB vs. +LPS	0.1225
3D	+LPS + BHB + AR vs. + LPS + BHB	0.3433
3D	+LPS + BHB + AR vs. + LPS	0.9495
3D	Control vs. + LPS	<0.0001
4A	+LPS + BH vs. + LPS	<0.0001
4A	+LPS + BHB + A vs. + LPS + BHB	0.9262
4A	+LPS + BHB + A vs. + LPS	0.0003
4A	+LPS + MCTB vs. + LPS + BHB + A	0.0022
4A	control vs. LPS	0.9992
4A	+LPS + BHB vs. + LPS	<0.0001
4B	-BHB + LPS – 1.5BHB + LPS	0.0072
5	IL-10: BHB + LPS – 1.0BHB + LPS	0.0466
5	IL-10: control vs. LPS	0.9090
5	TGF-β: BHB + LPS – 1.0BHB + LPS	0.0930
5	TGF-β: control vs. LPS	0.9239
5	CD206: BHB + LPS – 1.0BHB + LPS	<0.0001
5	CD206: control vs. LPS	<0.0001
S1A	Cell #: 0 mM BHB – 5 mM BHB	0.3432
S1A	ATP: 0 mM BHB – 5 mM BHB	0.8288
S1B	LPS vs. LPS + BHB	>0.9999
S1B	LPS + BHB vs. LPS + BHB + MCTB	>0.9999
S1B	LPS vs. LPS + BHB + MCTB	>0.9999
S1B	LPS + BHB + MCTB vs. LPS + MCTB	>0.9999
S1B	Control vs. MCTB	0.8237
S1C	-siRNA: BHB vs. -siRNA:+BHB	0.6598
S1C	-siRNA: BHB vs. +siRNA: BHB	0.9924
S1C	-siRNA: BHB vs. +siRNA:+BHB	0.9893
S1C	-siRNA:+BHB vs. +siRNA: BHB	0.8180
S1C	-siRNA:+BHB vs. +siRNA:+BHB	0.8354
S1C	+siRNA: BHB vs. +siRNA:+BHB	>0.9999
S2A	0 mM BHB – 5 mM BHB	<0.0001
S2A	0 vs. 0.16	0.4233
S2A	0 vs. 0.63	0.0004
S2A	0 vs. 1.25	<0.0001
S2A	0 vs. 2.5	<0.0001
S2A	0 vs. 5	<0.0001
S2B	Control vs. BHB	<0.0001
S2B	Control vs. BHB + MCTB	<0.0001
S2B	Control vs. MCTB	0.0048
S2B	BHB vs. BHB + MCTB	0.0434
S2B	BHB vs. MCTB	<0.0001
S2B	BHB + MCTB vs. MCTB	0.1404
S3	0 mM BHB vs. 0.1 mM BHB	0.5450
S3	0 mM BHB vs. 1 mM BHB	0.0264
S3	0 mM BHB vs. 5 mM BHB	0.0001
S4A	-siRNA -BHB vs. -siRNA + BHB	<0.0001
S4A	-siRNA -BHB vs. +siRNA + BHB	0.7372
S4A	-siRNA -BHB vs. +siRNA – BHB	0.0228
S4A	-siRNA + BHB vs. +siRNA + BHB	<0.0001
S4A	-siRNA + BHB vs. +siRNA – BHB	<0.0001
S4A	+siRNA + BHB vs. +siRNA – BHB	0.2437
S4B	-siRNA -BHB vs. -siRNA + BHB	0.0159
S4B	-siRNA -BHB vs. +siRNA + BHB	<0.0001
S4B	-siRNA -BHB vs. +siRNA – BHB	<0.0001
S4B	-siRNA + BHB vs. +siRNA + BHB	<0.0001
S4B	-siRNA + BHB vs. +siRNA – BHB	<0.0001
S4B	+siRNA + BHB vs. +siRNA – BHB	<0.0001

### Scope

This work fits well within the Frontiers in Aging scope because it attempts to uncover the molecular and cellular mechanisms underlying the previously observed longevity, memory, and cognitive benefits in aged and AD mouse models on a KD or BHB. We previously showed that KDs, but not control diets, improved memory in aged C57BL6 mice, and BHB levels uniquely increased on a KD, which suggests that BHB itself improved memory. Our other work showed that BHB was dose-dependently anti-inflammatory in *human* microglial cells, and this may explain previous *in vivo* observations. However, to understand the anti-inflammatory and pro-memory effect of the KD in *mice*, we tested whether BHB’s anti-inflammatory effect is concentration-dependent in a mouse microglial cell line. We showed that BHB was concentration-dependently anti-inflammatory in mouse microglial cells, and these anti-inflammatory effects were present precisely in the concentration range of BHB that produced positive therapeutic benefit in aged C57Bl6 mice, PS1/APP, and 5XFAD mice. This work suggests that BHB is the causative agent for the cognitive benefits we have observed in various mouse models of aging and AD and supports the view that maximizing BHB levels through a KD or with BHB supplements could support cognitive health span in humans.

## Data Availability

The raw data supporting the conclusions of this article will be made available by the authors, without undue reservation.
