# Supplementary figures and images for: Beta-hydroxybutyrate (BHB) elicits concentration-dependent anti-inflammatory effects on microglial cells which are reversible by blocking its monocarboxylate (MCT) importer
Source: Front Aging. 2025 Jul 29;6:1628835. doi: 10.3389/fragi.2025.1628835 (PMC12339548; doi:10.3389/fragi.2025.1628835)

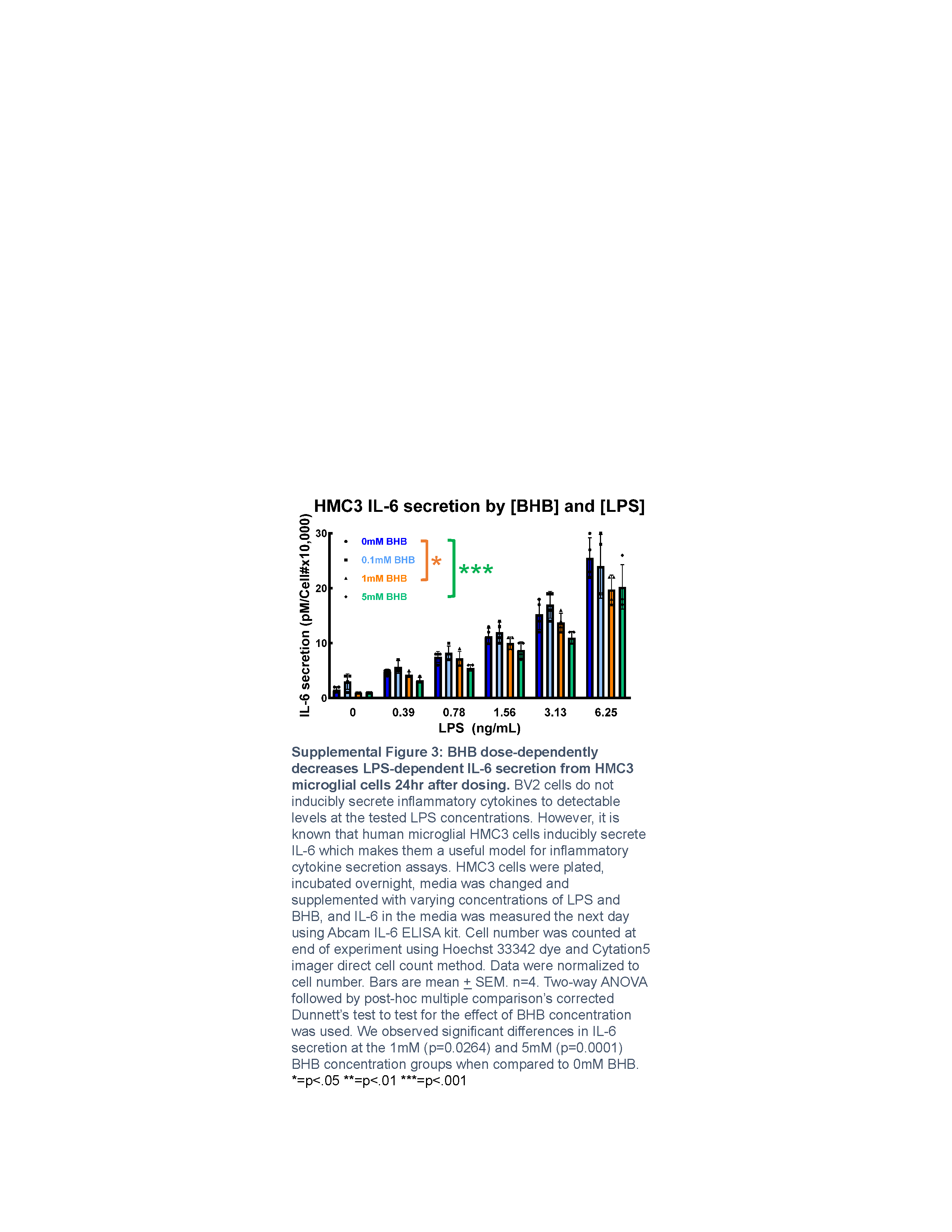

Supplement: Supplementary file 1 [file Image3.tif]

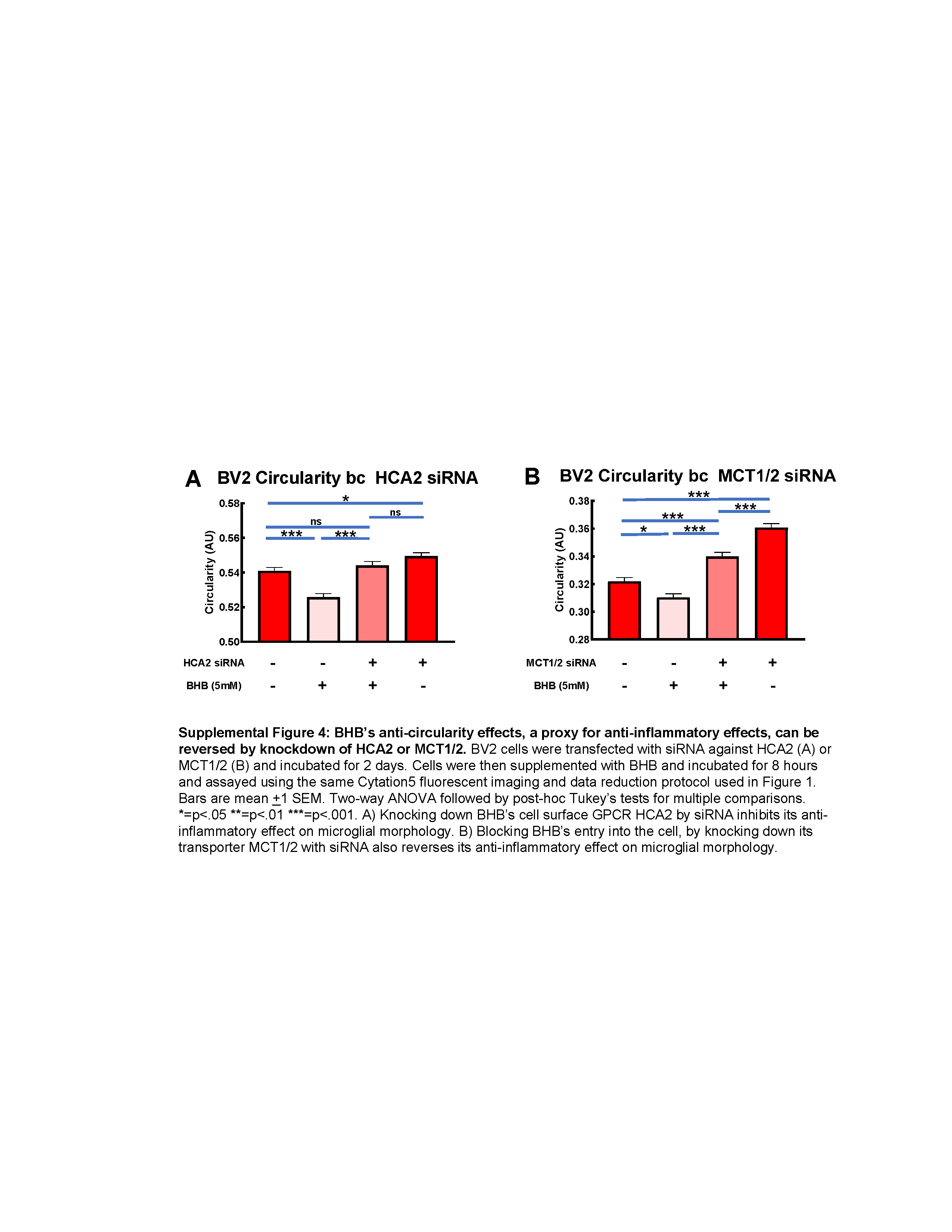

Supplement: Supplementary file 2 [file Image4.tif]

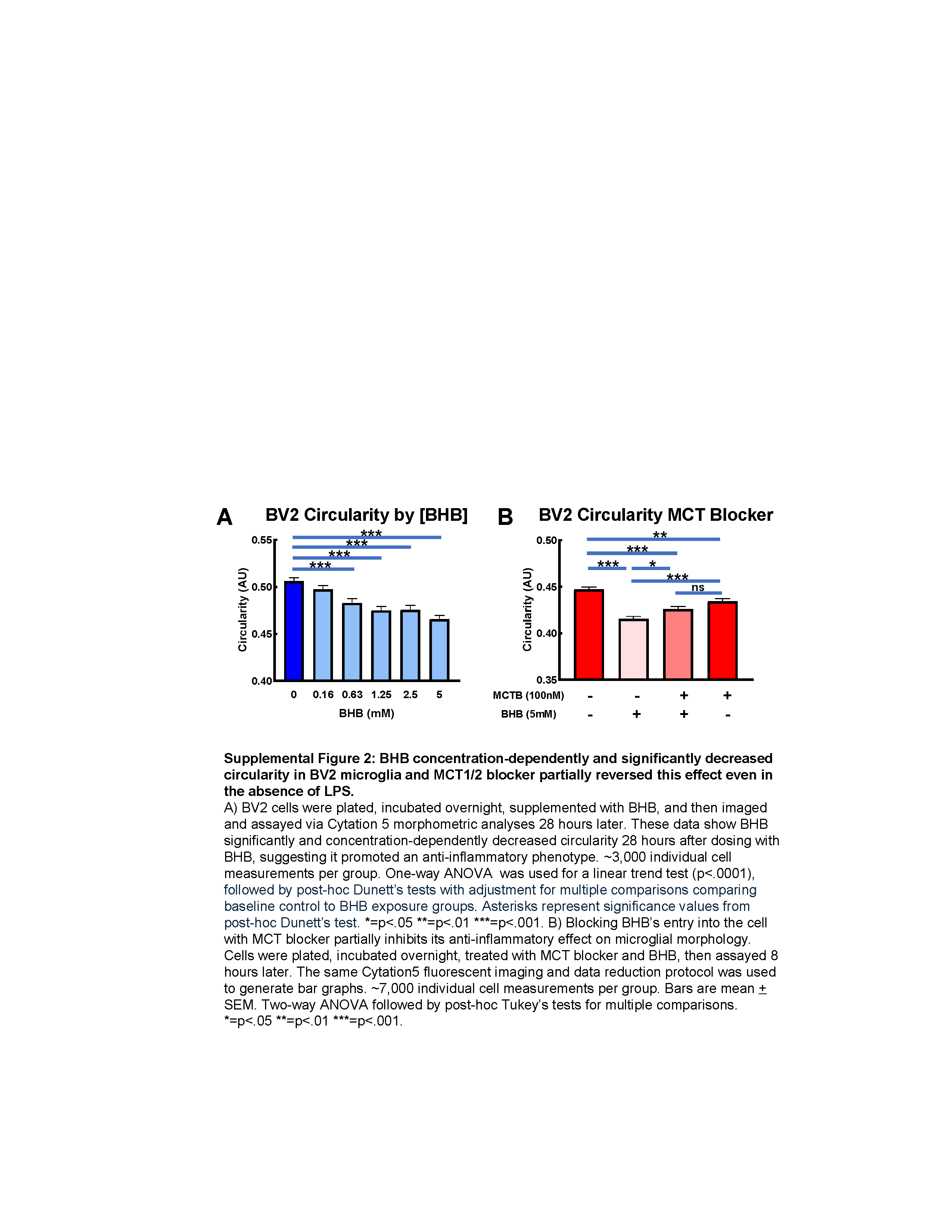

Supplement: Supplementary file 3 [file Image2.tif]

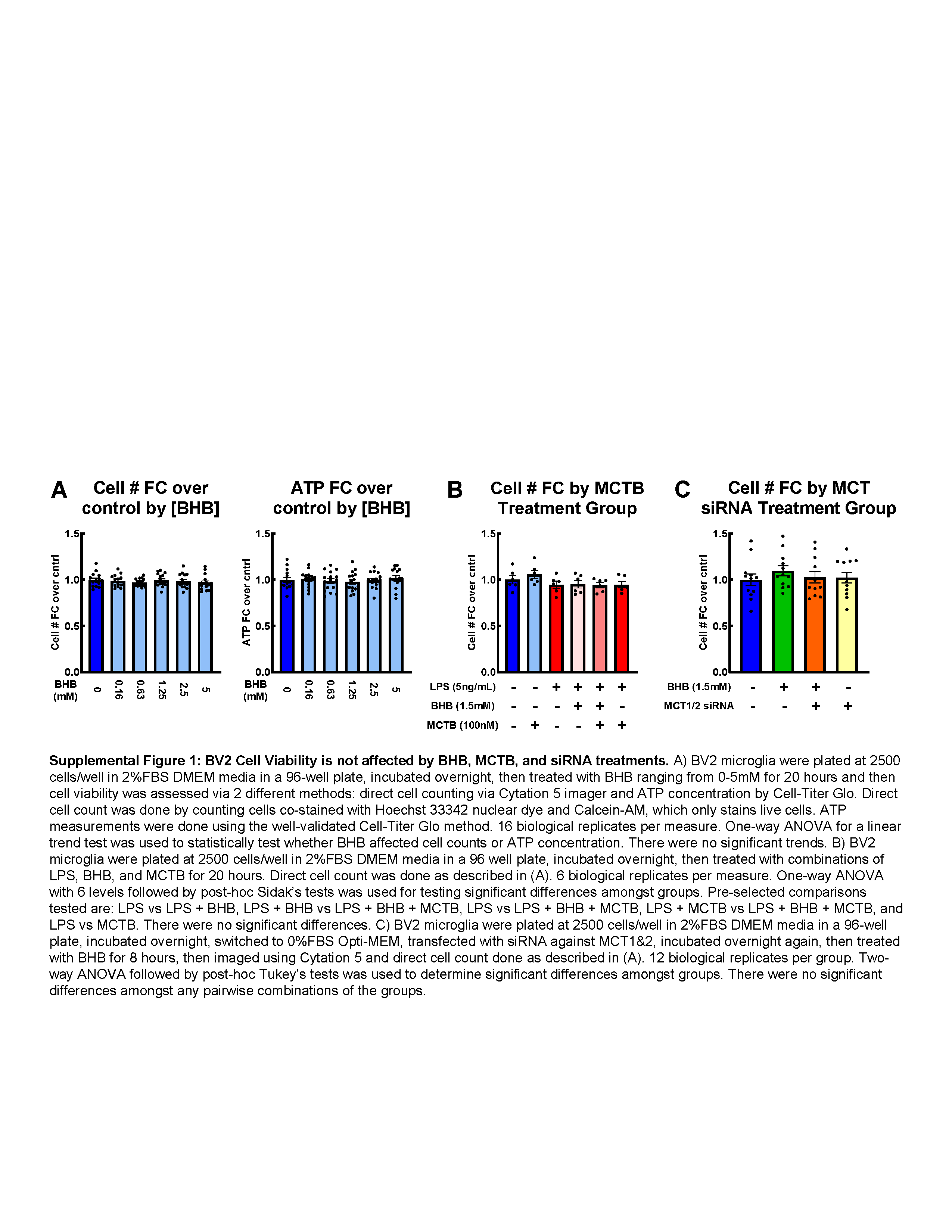

Supplement: Supplementary file 4 [file Image1.tif]
